# Recognition of systemic differences in municipal waste management in selected cities in Poland and the United States

**DOI:** 10.1007/s11356-023-27911-4

**Published:** 2023-06-03

**Authors:** Grzegorz Przydatek

**Affiliations:** Department of Engineering Science, University of Applied Sciences in Nowy Sącz, Zamenhofa 1a, Nowy Sącz, Poland

**Keywords:** Collection, Sorted and unsorted waste, Recovery, Accumulation, Indicator, Model autoregressive integrated moving average (ARIMA)

## Abstract

This study aims to demonstrate differences in the efficiency of municipal waste management from 2014 to 2017 between two selected cities with a comparable number of inhabitants: Radom in Poland and Spokane, WA, in the United States. The study considers the significance of these cities’ rates of waste accumulation and the application of the autoregressive integrated moving average model for forecasting. Within a 4-year period, Spokane recorded a higher total mass of waste collected (4175.4 Mg) than Radom, while Radom recorded a higher monthly average (exceeding 500 Mg) than Spokane. In these cities, nonselectively collected waste was predominant, with an average mass of 1340 Mg, and the highest accumulation rate per capita in the European Union was recorded in Radom (174.04 kg per year). An increase in the number of residents by 2000 people in Spokane fostered an increase in waste accumulation rates per capita by an average of more than 11 kg per year, with the highest value of selectively collected waste accumulation per capita reaching 102.18 kg per year. In comparison to Radom, the Spokane city waste management system is characterised by projected waste growth, greater efficiency, a higher accumulation of selective waste, and rational waste to energy processing. Generally, the results of this study indicate a need to develop rational waste management, while taking into account the principles of sustainable development and the requirements of the circular economy.

## Introduction

In recent years, urbanisation has increased globally, and consequently, the amount of waste produced has also increased (Aslam et al. 2020). Waste generation is often directly related to urbanisation, lifestyle, and affluence (OECD 2007; Ogbonna et al. 2007). Effective waste strategies depend on local solutions and social awareness. According to Matsakas et al. (2017), the accumulation of waste in the environment raises social awareness.

Waste management is a very complex process that involves a wide range of tasks: waste prevention, production, collection, transport, recycling, processing, and many others (Smejkalová et al. 2020). It is becoming increasingly important, because rational waste management is associated with the minimisation of waste and its adverse environmental effects.

In the United States (US), waste management objectives are included in state solid waste management plans, the objectives of which are set out in the Act 4002(b) (n.d.). On this basis, individual states, including Spokane County, require reduction of the total amount of waste generated per person and the production of energy from its processing (PWM 2021). In the European Union (www.ec.Europa.EU), the management, treatment, and disposal of waste are regulated by, among others, directives on landfilling (Directive 1999/31/EC), waste incineration (Directive 2000/76/EC), and waste in general (Directive 2008/98/EC). These establish a legal framework for waste management in EU member states which governs a hierarchy (Kostecka et al. 2014) and the effective use of installations for “recovery” rather than “disposal” (Grosso et al. 2010). In Poland, the relevant guidelines (Act 1996, 2001) oblige local governments to develop systems for selective waste collection and, at the same time, to limit waste storage with possible incineration.

However, the EU and its member states are taking measures to apply the waste hierarchy to encouraging solutions that minimise environmental influence (Pomberger et al. 2017). In the hierarchy of waste harmlessness, landfilling is classified as a form of waste disposal. The burning of waste is regarded as a method of making economic savings. Further to this, Biernat et al. (2011) described a system in the US that produces electricity from the disposal of waste. Wang et al. (2021) concluded that the US produces the greatest mass of municipal waste worldwide, whereas Poland ranks 26th in the EU in this respect (www.ec.Europa.EU).

Kollikkathara et al. (2009) emphasised the importance of using waste accumulation indicators to assess the effectiveness of waste management on a global scale. Kamaruddin et al. (2017) also used such waste accumulation rates. Some researchers (Przydatek 2020; Przydatek and Ciągło 2020) have demonstrated the diversity of waste accumulation indicators in use in Poland, considering both urban and rural waste management. Urban waste management has been the subject of increasing attention because excessive amounts of garbage remain uncollected in the streets, causing inconvenience, environmental pollution, and public health risks (Yoada et al. 2014).

The current waste management trend is shifting from the principles of a classical linear economy to those of a circular economy (Prieto-Sandoval et al. 2018). Ultimately, a circular economy is a project of resource transformation, and the distribution, use, and recovery of goods and materials require planning and forecasting (Stahel 2016). According to Nęcka et al. (2019), municipal waste management planning requires, among other things, reliable forecasts of accumulated waste. According to World Bank data, the quantity of waste produced globally is expected to grow to 3.40 billion tonnes by 2050 (www.datatopics.worldbank.org).

The prediction of waste mass is therefore an essential element of waste management (Cubillos 2020). On this basis, some researchers (Gaska et al. 2018) have focused particular attention on using modelling to optimise waste management; this includes planning collection paths, designating recycling points and collection yards, and other actions. One of the best-known and reliable forecasting methods in waste management is the autoregressive integrated moving average (ARIMA), which is based on regression (Sen et al. 2016). Researchers such as Chauhan and Singh (2017) have applied the ARIMA model to waste management.

This study aims to demonstrate the differences between the efficiency of municipal waste management in two selected urban units in Poland and the US with a comparable number of inhabitants in the period 2014–2017, and to consider the importance of accumulation and prediction in these two urban units (United States Census Bureau 2014).

## Materials and methods

The data used in this study are based on questionnaires addressed to municipalities in Radom, Poland, and Spokane, WA, US. Based on monthly data covering the period from 2014 to 2017, the data include the total quantity of waste selectively and nonselectively collected. The analysis of these data included determination of the mass accumulation indicator per capita for Radom and Spokane. Studies by Kawai and Tasaki (2016) and Przydatek (2016) have previously used waste accumulation as an indicator. The data used in the present study include the following: the annual number of inhabitants in the cities of Radom (Statistics Poland 2014–2017) and Spokane (US Census Bureau 2014–2017), and monthly/annual data on the waste generated in these cities, which was broken down into nonsegregated and segregated waste and the total mass of waste (Municipalities of Radom and Spokane).

The data on collected waste were subjected to statistical analyses considering the following descriptive statistics: minimum, maximum, mean, and standard deviation. Extreme values, mean values, and standard deviations are presented using box plots for selected elements that differ significantly from one point to another. The analysis of variance (ANOVA) test was used to assess whether differences in the waste accumulation mass between the selected cities were statistically significant. As most of the variable decomposition conditions were met (except for segregated waste in Radom), the significant differences between the masses of the waste collected in each city were estimated, and Student’s parametric *t*-test (for independent samples) was used to compare the two independent groups. The *F*-test was also used to test for significant differences between the two variances (Przydatek 2021).

Malska and Wachta (2015) demonstrated that a specific numerical value resulting from the prediction process is a forecast. Other researchers (Du et al. 2014) have pointed to developing research based on predictive results. In the present study, to forecast the amount of unsorted and segregated waste, a time series was developed based on data for the two selected cities using the ARIMA model. It was created by combining the autoregressive model with a moving average and using differentiation. The study specifies models of the development tendency and presents forecasts for the future. A critical analysis of the literature revealed that ARIMA models are considered the most accurate method for forecasting time series with trends and seasonal fluctuations (Kozicki et al. 2019).

In this study, the significance level was set as *p* < 0.05, and for the ARIMA model, trends were set at the level of *p* < 0.1. The number of data points (i.e. the monthly sum of waste mass over the 4-year period) used for analytical purposes for each of the six variables was *n* = 48. Statistica 13 (StatSoft Poland, StatSoft, Inc., USA) software was used to conduct the statistical analyses.

## Descriptions of the selected cities

### Radom

**Fig. 1:**
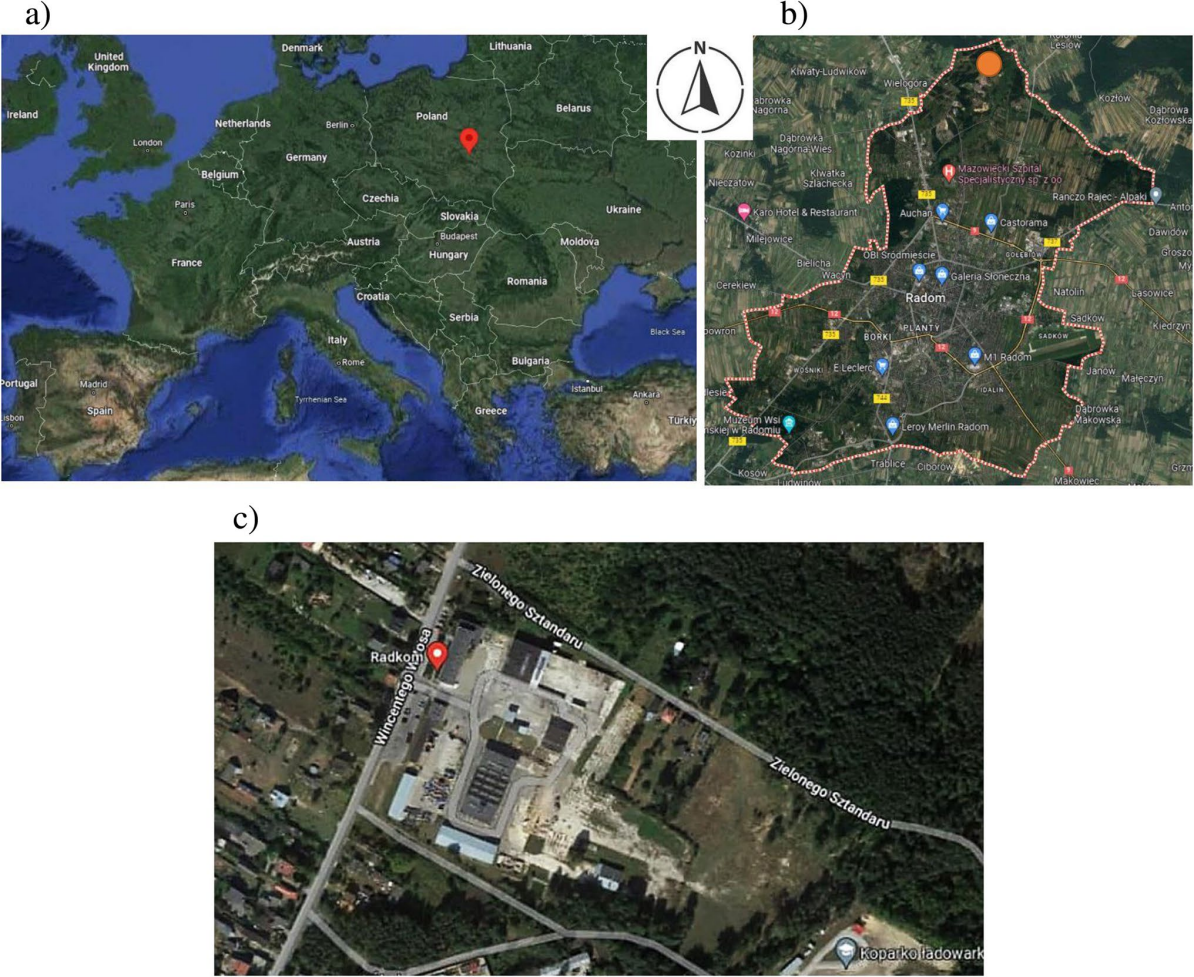
**a**) Localisation of Radom city in central-eastern Poland (southern of the Mazowieckie Voivodeship). **b**) Localisation of the regional installation for waste management in the northern part of Radom city. **c**) The area of the regional waste management installation in Radom

The city of Radom is situated in central-eastern Poland in the southern part of the Mazowieckie Voivodeship, 100 km from the capital of Warsaw, at 21°09′24″E longitude and 51°24′13″N latitude and at an altitude of 130 to 207 m above sea level (Fig. 1a). The city covers an area of 111.8 km^2^. Radom is the second largest city in the Mazowieckie Voivodeship.

The administrative divisions of Radom include 61 districts and housing estates. Radom is famous primarily for its modern metal industry. The city produces various machine parts produced by precision engineering. Some companies operating in the city produce automobile components and electrotechnical devices (Program 2021).

### Spokane

**Fig. 2:**
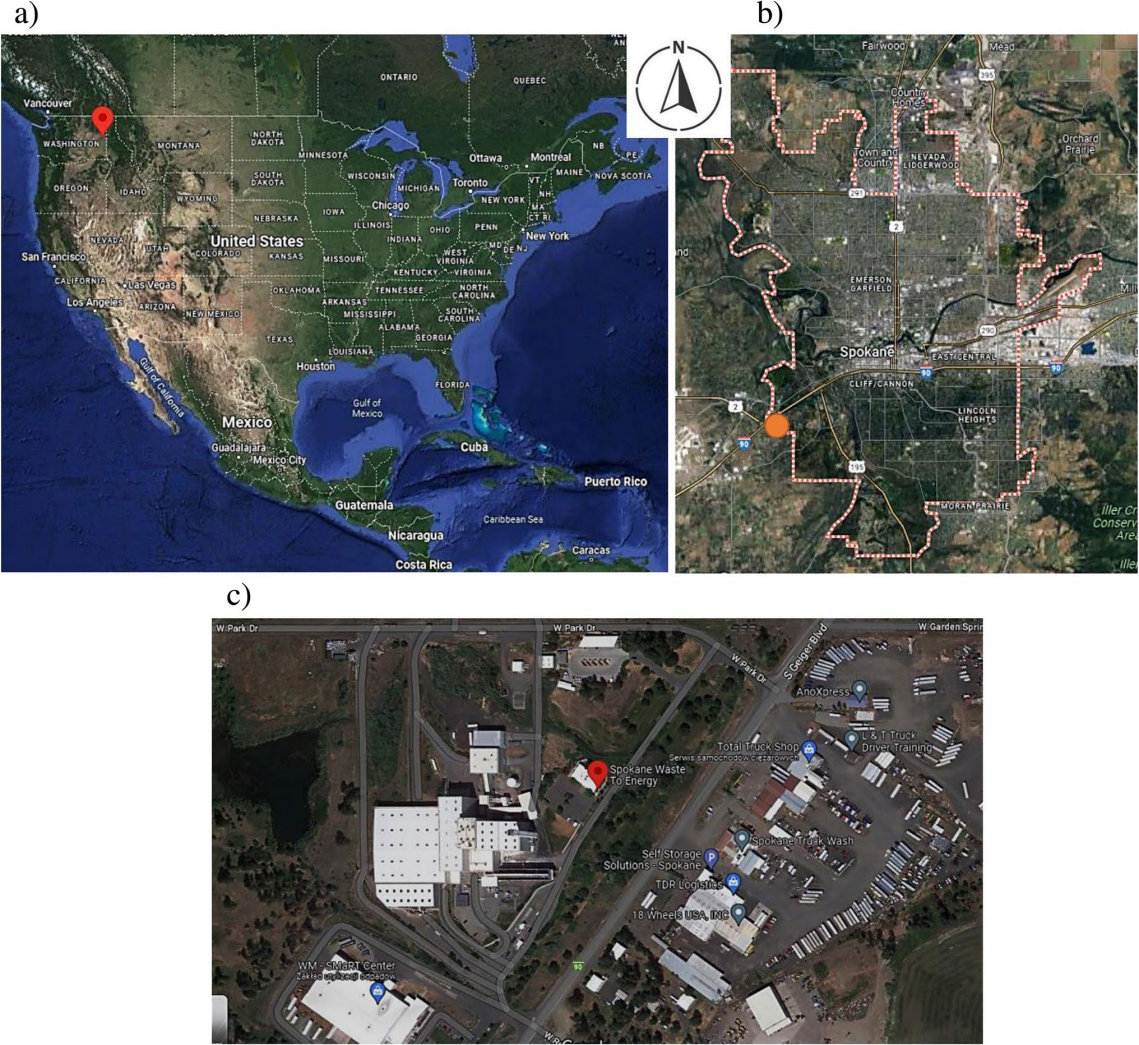
**a**) Localisation of Spokane city in north-western of the United States (Eastern Washington). **b**) Localisation of the WtE installation in the south-western part of Spokane city. **c**) The area of waste to energy plant in city of Spokane

Spokane is the largest city and seat of Spokane County, WA. It is situated east of the Spokane River, adjacent to the Selkirk Mountains and west of the foothills of the Rocky Mountains, 148 km south of the Canadian border. Spokane is located at 117°25′30″ longitude and 47°39′32″N latitude and at an altitude of 724 m above sea level (Fig. 2a). The city occupies an area of 151.6 km^2^. The Spokane region has warm, dry summers and cool, moist winters.

Spokane has become an important railroad and shipping centre due to its location between mining and agricultural areas. The leading manufacturing sectors are wood and food processing, printing and publishing, metal refining and manufacturing, the production of electrical and computer equipment, and the production of transport equipment. Mining, forestry, and agribusiness remain essential to the local and regional economy, but Spokane’s economy is diverse and includes other industries, including high-tech and biotechnology industries (www.pubs.usgs.gov).

## Waste management

### Radom

In Radom, solid municipal waste (MSW) is collected weekly, and bulky waste and used electrical and electronic equipment are collected every 3 months. MSW is collected both selectively and nonselectively. The selective collection of waste is based on the following categories: paper, glass, metals, organics, bulky, hazardous, and ash (www.radom.pl).

Nonselective and selective municipal waste is transferred for management at the municipal waste disposal plant, located in the northern part of the city (Fig. 1b). This regional installation includes a mechanical–biological waste treatment installation, a large waste processing line, a line for processing construction and similar waste, and an installation for the storage of waste generated in the process of mechanical and biological treatment of unsorted municipal waste and residues from sorting municipal waste. The installation receives waste from Radom and over 60 communes in the Radom region (Program 2021; Fig. 1c).

On the premises of this plant, there is a municipal selective waste collection point that accepts selectively collected domestic municipal waste, which is collected separately.

### Spokane

The waste generated by Spokane is collected weekly. Waste is collected both selectively and nonselectively. The following types of waste are collected selectively: paper, plastics, glass, metals, hazardous waste, bulky waste, and demolition waste (www.library.municode.com).

The mixed waste management system is mainly based on waste to energy (WtE). Spokane operates Washington’s only WtE facility along with two solid waste transfer stations as part of the Spokane Regional Solid Waste System, a partnership between Spokane and Spokane County. The plant is located in the south-western part of the city (Fig. 2b).

Spokane owns and operates a waste disposal facility for the Spokane County Regional Solid Waste System (Fig. 2c). The solid waste is incinerated to produce steam which is then used to generate electricity via a turbogenerator. The energy is mainly used to operate the facility (www.spokanecounty.org).

## Results

The analysis of the results revealed that the highest average value for total waste collected in Spokane was 4175.40 Mg, with minimum and maximum values of 3353.64 and 5032.09 Mg. The highest average amount of separately collected waste in Spokane in the research period was 1693.44 Mg, which was 788.52 Mg less than the average amount of nonselectively collected waste in Spokane in the same period. The lowest average amount of segregated waste collected was in Radom (1109.39 Mg) and was lower than the average amount of unsorted waste collected by 1892.35 Mg. In Radom, the highest standard deviation was recorded (561.29 Mg for total collected waste), with the lowest standard deviation (144.91 Mg) observed in Spokane (for segregated waste; see Table 1).

**Table 1:** Descriptive statistics of the waste collected in Radom and Spokane

Variability	Descriptive statistics				
	Nimportant	Average (Mg)	Min. (Mg)	Max. (Mg)	Standard deviation (Mg)
Spok_mixed_waste	48	2481.96	1967.58	3088.48	278.32
Spok_segreg_waste	48	1693.44	1349.91	1994.26	144.91
Spok_total_amount_waste	48	4175.40	3353.64	5032.09	374.98
Rad_mixed_waste	48	3001.74	2393.86	3511.86	244.02
Rad_segreg_waste	48	1109.39	382.56	1891.82	457.28
Rad_total_amount_waste	48	4128.71	2974.33	5330.82	561.29

**Fig. 3:**
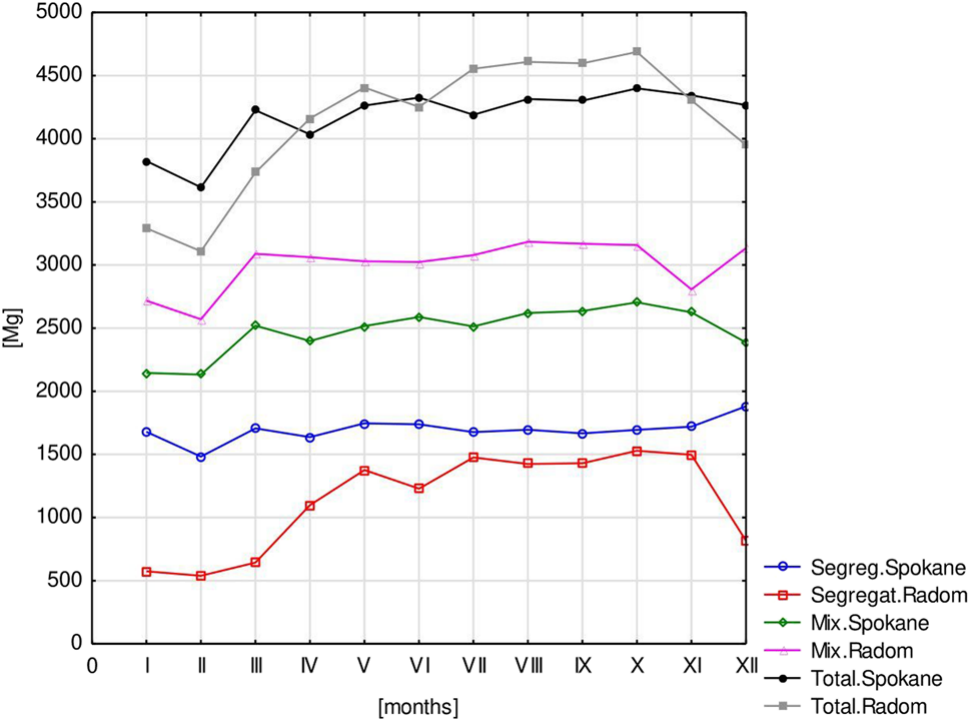
The average amount of unsorted waste collected in Spokane and Radom

In the monthly breakdown, the lowest average amount of waste collected (3108.39 Mg) and the highest average amount of waste collected (4688.03 Mg) were in recorded in Radom in February and October. The highest average amount of segregated waste collected (1880.38 Mg) was recorded in Spokane (XII), and the lowest (538.78 Mg) was recorded in Radom (II). The average amount of unsorted waste collected ranged from 2131.75 Mg (II) in Spokane to 3184.21 Mg (VIII) in Radom (Fig. 3).

In Radom, the number of inhabitants decreased noticeably by over 2000 over the study period, with the highest population recorded in 2014 at 217,201 and the lowest in 2017 at 214,566. Similarly, the highest accumulation rate of unsorted waste per capita (174.04 kg) was recorded in the first year. This result confirms a decrease of 12 kg capita^−1^ year^−1^ over the 4-year study period. In contrast, the highest value of this indicator for segregated waste was 79.05 kg capita^−1^ year^−1^ in 2017, an increase of about 36 kg capita^−1^. In Spokane, the population grew by more than 2000 inhabitants during this period, with the highest population of 212,982 recorded in 2017 and the lowest in 2014 at 210,142. Waste accumulation indicators also show an increase in unsorted waste accumulation by more than 10 kg capita^−1^ year^−1^ and a greater increase in segregated waste accumulation of 12.37 kg capita^−1^ year^−1^. The highest amounts of unsorted and segregated waste collected were 151.22 and 102.18 kg capita^−1^ year^−1^, respectively, achieved in the last audited year when the population was at its lowest (Table 2).

**Table 2:** Waste accumulation rates per average inhabitant of Radom and Spokane city

Indicators/waste	Nonsegregated				Segregated			
	2014	2015	2016	2017	2014	2015	2016	2017
Radom								
Inhabitants	217,201	216,159	215,02	214,566	217,201	216,159	215,02	214,566
Waste accumulation (kg cap^−1^ year^−1^)	174.04	165.73	166.00	162.02	43.19	55.83	74.99	79.05
Spokane								
Inhabitants	210,142	210,695	212,078	212,982	210,142	210,695	212,078	212,982
Waste accumulation (kg cap^−1^ year^−1^)	140.91	128.18	142.92	151.22	89.81	94.29	98.00	102.18

### Statistical analyses

**Fig. 4:**
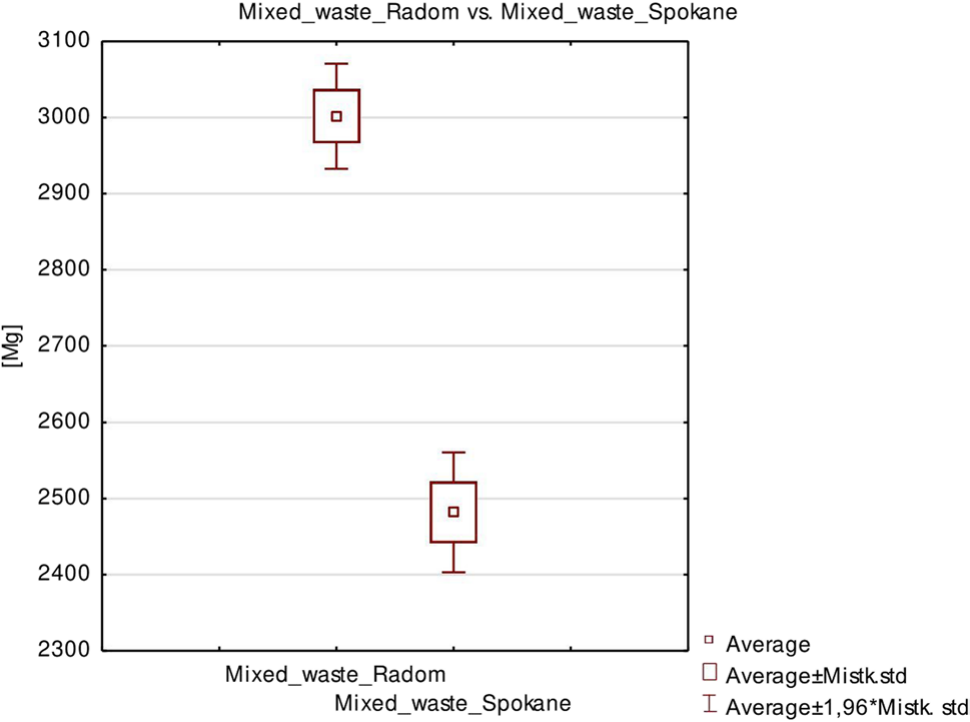
Comparative analysis of the collected waste using Student’s *t*-test and the *F*-test

A comparative analysis of the collected waste using Student’s *t*-test and the *F*-test revealed that the amount of waste collected did not differ significantly between the two studied cities (Fig. 4), which rules out the homogeneity of the variance of the excluded studies. Thus, the uncontrolled amount of collected waste in Radom was statistically marked.

**Fig. 5:**
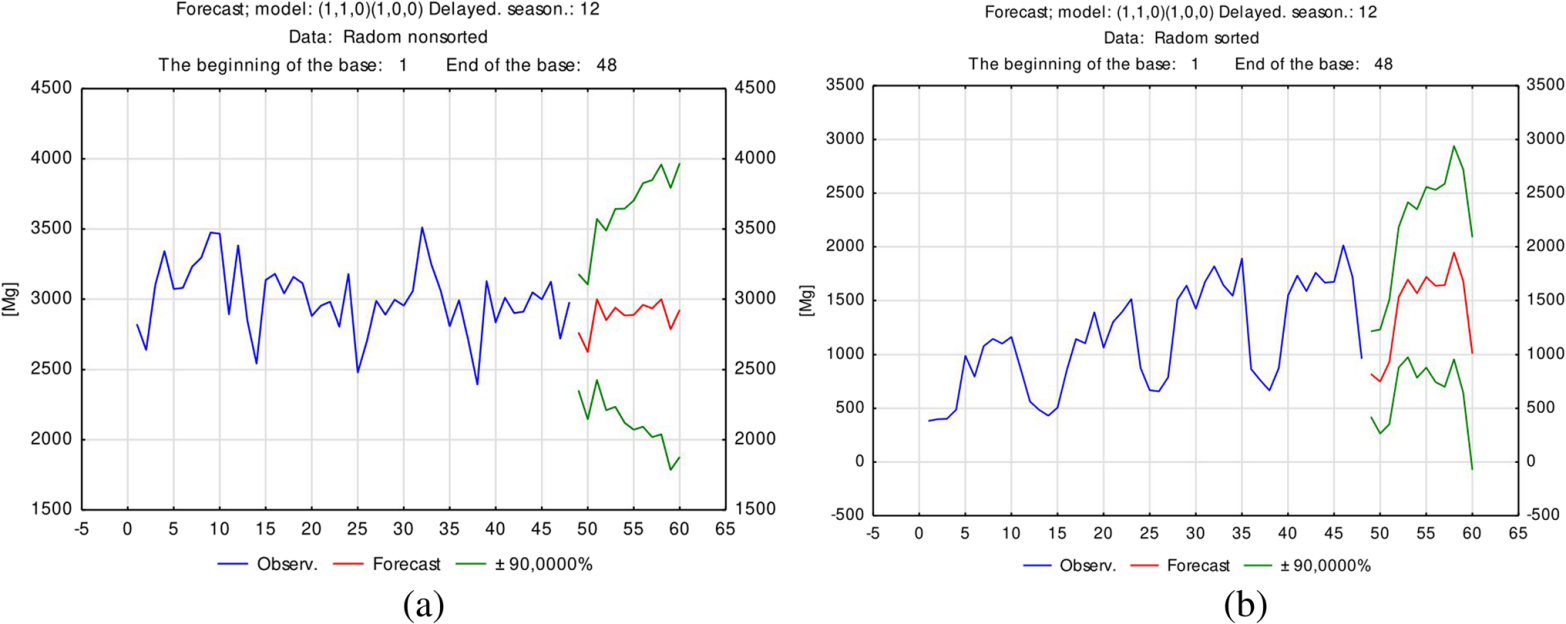
Forecasting results for the amount of segregated and unsorted waste in Radom

As shown in Fig. 5, the forecasting results for the amount of segregated (a) and unsorted (b) waste in Radom in the next period do not exhibit an upward trend.

**Fig. 6:**
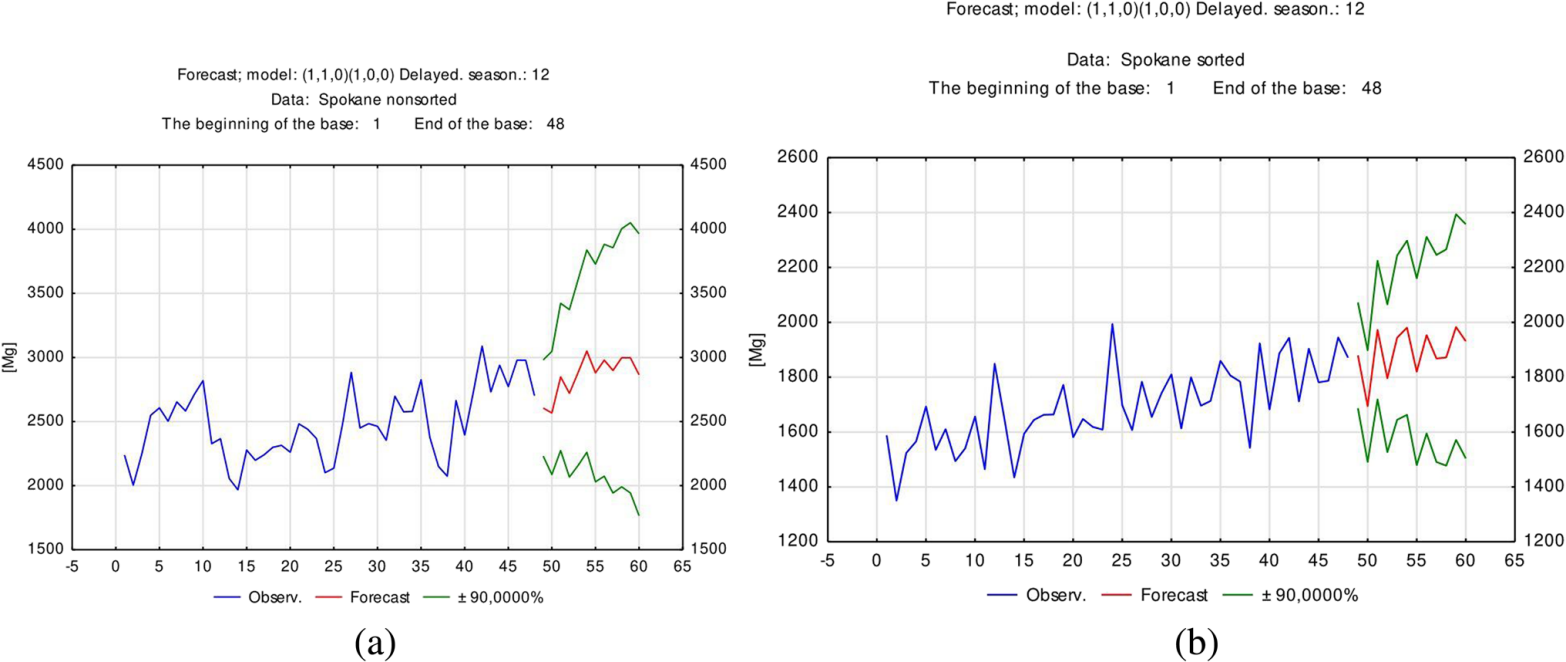
Forecasting results for the amount of unsorted and segregated waste in Spokane

Analysis of the forecasting results shown in Fig. 6 confirmed that the amount of unsorted (a) and segregated (b) waste in Spokane in the next period exhibits an upward trend; this is confirmed at the significance level of *p* < 0.1.

## Discussion

The production of solid waste is an inevitable consequence of human activity and is especially noticeable in cities (Vergara and Tchobanoglous 2012). In the two selected cities, two population trends are noticeable. The increase in the population of Spokane city, which also recorded the highest average waste of 4175.40 Mg, by 2000 people should be considered beneficial. Such an increase may affect urban development (Mesjasz-Lech 2014), as increases in waste generation are associated with increases in population (Kijewska et al. 2012).

In the analysed urban centres, the prevalence of nonselectively collected waste in a range from 788.52 to 1892.35 Mg is noticeable. Mazurkiewicz (2018) demonstrated a similar condition in Poland more generally. These differences indicate the need to develop the collection of separated waste.

The highest average amounts of total waste collected (4688.03 Mg) and unsorted waste collected (3184.21 Mg) occurred in Radom in the autumn and summer, respectively, which confirms the seasonal variability of waste collection. According to Tchobanoglous and Kreith (2003), the composition of generated waste is highly variable due to seasonal, lifestyle, cultural, demographic, geographic, and legal influences. There was a significantly higher amount of nonselectively accumulated waste in Radom compared to selectively accumulated waste. The dominance of unsorted waste in the waste management system is conditioned by specific problems with the implementation of regulations governing waste management in the selected cities in Poland (Act 1996) and the US (Act 42 U.S.C. 6942(b) n.d.), and by the insufficient social awareness of the cities’ residents (Guerrero et al. 2013; Scheinberg et al. 2010).

Per capita waste accumulation data are widely used to compare the intensity of MSW generation at various sites (Karak et al. 2012). The highest accumulation rate of unsorted waste per capita (174.04 kg per year) was recorded in Radom when the city’s population was at its highest, and was higher by 22.82 kg per year than in Spokane. Guerrero et al. (2013) showed a relationship between the amount of waste and the number of inhabitants. On the other hand, Przydatek (2020) found a higher rate by half than the waste accumulation rate per statistical inhabitant of Poland, and Thorneloe et al. (2008) revealed that this indicator was over three times higher for an average-sized city in the US (750,000). Spokane recorded the highest accumulation of segregated waste per capita (102.18 kg year^−1^) when its population was at its lowest over the study period, and this figure was higher by 23.13 kg compared to that obtained in Radom. This indicates that the efficiency of separate collection also depends on various structural economic factors (Passarini et al. 2011). The two considered waste accumulation rates per capita were found in the US, with an average increase of over 11 kg capita^−1^ year^−1^. Felice (2014) concluded that the accumulation of household solid waste is the first step in the chain of its municipal management.

Generally, Spokane exhibited an upward trend in generated waste over the study period, collected both selectively and nonselectively. Similarly, Pattnaik and Reddy (2010) found an increase in generated waste in India. Kumar et al. (2009) noticed a strong correlation between generated waste and population growth. In addition, Kollikkathara et al. (2009) linked such growth to economic development. In the present study, the forecast based on the statistical data analysis of the two cities also confirmed a prospective increase in the waste mass in the US, indicating that Spokane will experience an increase in waste generated per capita instead of the expected reduction through sustainable materials management (PWM 2021). According to Singh and Ordoñez (2016), using such statistical tools is very helpful for the interpretation of datasets.

Differences in waste management between the two municipalities were also evident in the present study. In Radom, unsorted and sorted municipal waste is transferred to the regional waste processing plant, which consists of a sorting plant, a composting plant, and a waste landfill site. Laner et al. (2012) suggested that, despite the measures taken to minimise the mass of generated waste and ensure its rational recovery, landfilling remains the most widely used waste management solution worldwide. It should be noted that a society that strives for long-term sustainability requires long-term, sustainable landfills (Vaverková 2019).

In Spokane, waste is transported to the WtE facility through two solid waste reloading stations, which operate under the Spokane Regional Solid Waste System. This system also covers the storage of residues. Moya et al. (2017) emphasised that WtE technologies are practical industrial solutions for converting large amounts of MSW into energy sources. Cucchiella et al. (2014) confirmed that incineration is the best option when considering cogeneration. For Spokane, the conversion of waste into energy is a prerequisite for a circular economy. The lack of a solution for thermal waste treatment in Radom indicates a possible need for increased waste storage capability, and a life cycle assessment (LCA) could help the city to achieve sustainable development goals (Pujara et al. 2019). On the other hand, such a state indicates that it may be possible to limit the landfilling of municipal waste through the use of a hybrid incineration-gasification system using Refuse Derived Fuel (RDF) (Kerdsuwan et al. 2015). Research (Malinauskaite et al. 2017) indicates that this may minimise the amount of waste deposited in landfills.

The results of the present study indicate that differences in MSW in the two studied cities, which have similar populations, are linked by the dominance of unsorted waste, which confirms the need to strengthen activities to maintain the principles of sustainable development, and in particular to maintain the hierarchy of waste management both in the EU and the US, in accordance with the 3R principle (Reduce, Reuse and Recycle) (Pactwa et al. 2020).

## Conclusion

Based on the comparative analysis of waste management in two selected cities in Poland and the US conducted from 2014 to 2017, it can be concluded that, in Spokane, the highest monthly average amount of total waste was 4175.40 Mg, whereas the highest monthly average in Radom was in the autumn at 4688.03 Mg, with nonselectively collected waste being more prevalent by an average of 1340 Mg. On the other hand in Radom, a significantly higher amount of nonselectively accumulated waste and the highest accumulation rate of this waste per capita of 174.04 kg year^−1^ indicate the need to develop selective waste collection. In Spokane, the segregated waste accumulation rate per capita of 102.18 kg year^−1^ when the population was at its lowest was 23.13 kg higher than in Radom. A noticeable increase of 2000 in the population of Spokane fostered an increase in waste accumulation rates per capita of an average of more than 11 kg^−1^ capita^−1^, which is in line with the forecast for an increase in waste accumulation in this area and indicates the need to strengthen waste reduction activities. In this city, waste management system is more efficient than the system in Radom due to the efficient conversion of waste into energy, which is an integral part of the circular economy.

In the two analysed cities, the highest average amount of waste accumulated over the 4-year study period was recorded in Spokane, and the highest monthly average was recorded in Radom. In both cities, nonselectively collected waste was more prevalent, with the highest accumulation rate per capita in Radom. The waste management system in Spokane is characterised by greater efficiency of waste accumulation using selective collection and by rational processing of the residues into energy, thus strengthening the circular economy. It is advisable to strengthen the rational handling of waste in order to maintain sustainable development in the studied cities.

## Data Availability

The datasets used and/or analysed during the current study are available from the corresponding author on reasonable request.
